# Non-Union Treatment in the Foot, Ankle, and Lower Leg: A Multicenter Retrospective Study Comparing Conventional Treatment with the Human Allogeneic Cortical Bone Screw (Shark Screw^®^)

**DOI:** 10.3390/jpm14040352

**Published:** 2024-03-27

**Authors:** Viktor Labmayr, Elisabeth Huber, Florian Wenzel-Schwarz, Patrick Holweg, Martin Ornig, Gerd Jakob, Wolfgang Palle, Gudrun H. Borchert, Klaus Pastl

**Affiliations:** 1 Department of Orthopaedics and Trauma, Medical University Graz, Auenbruggerplatz 5, A-8036 Graz, Austria; p.holweg@medunigraz.at (P.H.); martin.ornig@uniklinikum.kages.at (M.O.); 2 DOKH Friesach, St Veit Str. 12, A-9360 Friesach, Austria; elisabeth.huber@krakenhaus-friesach.at (E.H.); wolfgang.palle@dokh.at (W.P.); 3 Orthopädisches Spital Speising Wien, Speisinger Straße 109, A-1130 Wien, Austria; florian.wenzel-schwarz@oss.at; 4 Landeskrankenhaus Villach, Nikolaigasse 43, A-9500 Villach, Austria; gerd.jakob@kabeg.at; 5 Dr. Borchert Medical Information Management, Egelsbacher Str. 39e, D-63225 Langen, Germany; gudrun.borchert@borchert-medical.com; 6 Klinik Diakonissen Linz, Weißenwolffstraße 13, A-4020 Linz, Austria; klaus@pastl.at

**Keywords:** bone healing, non-union, human allogeneic cortical bone screw, Shark Screw^®^, pseudarthrosis, foot and ankle, time to union, delayed union, hardware removal, health care costs

## Abstract

Addressing non-unions involves stabilizing the affected area through osteosynthesis and improving bone biology using bone grafts. However, there is no consensus on the optimal treatment method. This study aims to compare outcomes of non-union surgery using conventional treatment methods (metal hardware ± graft) versus osteosynthesis with the human allogeneic cortical bone screw (Shark Screw^®^) alone or in combination with a metallic plate. Thirty-four patients underwent conventional treatment, while twenty-eight cases received one or more Shark Screws^®^. Patient demographics, bone healing, time to bone healing, and complications were assessed. Results revealed a healing rate of 96.4% for the Shark Screw^®^ group, compared to 82.3% for the conventionally treated group. The Shark Screw^®^ group exhibited a tendency for faster bone healing (9.4 ± 3.2 vs. 12.9 ± 8.5 weeks, *p* = 0.05061). Hardware irritations led to six metal removals in the conventional group versus two in the Shark Screw^®^ group. The Shark Screw^®^ emerges as a promising option for personalized non-union treatment in the foot, ankle, and select lower leg cases, facilitating effective osteosynthesis and grafting within a single construct and promoting high union rates, low complications, and a rapid healing process.

## 1. Introduction

Bone healing is a complex, scarless process critical for tissue restoration following a fracture or a surgical procedure like osteotomy and arthrodesis. Non-union is a deviation in the bone healing process [1,2]. It is attributed to patient-dependent factors—such as age, gender, comorbidities like diabetes mellitus, obesity, neuropathy, vitamin D deficiency, infections, smoking, alcoholism, and the use of non-steroidal anti-inflammatory drugs—or patient-independent factors like inadequate stability, poor blood supply, fracture type and location, gap width, soft tissue damage, bone defects, prior surgery, and the quality and type of osteosynthesis [1,2,3,4,5,6,7,8,9]. Clinical signs of the non-union of the lower extremity are pain [10] that is exacerbated during weight bearing, often accompanied by localized swelling [3,4] and reduced functionality [11].

Addressing non-unions entails stabilizing the affected area through stable mechanical fixation while providing a supportive biological environment and scaffold for bone healing through grafts [4,12,13,14,15]. However, using autologous grafts leads to donor site morbidity with postoperative pain, scarring, seroma, sensory loss, risk of infection, and longer time of surgery [16,17,18,19]. However, evidence exists showing that allograft use results in similar bone healing as when using autografts [20,21,22,23,24]. In practice, a spectrum of surgical principles, including various combinations and variations, are applied [7,25,26,27,28,29]. There is no consensus over the optimal treatment method [4].

The Shark Screw^®^ represents an alternative solution for non-union treatment, utilizing human allogeneic cortical bone with a threaded design and a diameter of up to 5.0 mm [30]. Engineered for stable osteosynthesis, it can be used independently or in conjunction with metal plates or screws. The Shark Screw^®^ merges human cortical bone properties with screw stability, addressing non-union surgery principles by integrating mechanical and biological aspects [4,13,27]. The allogeneic cortical bone screw demonstrated consistent success in fracture and osteotomy fixation [31,32,33], primary arthrodesis [34,35], salvage arthrodesis [36], and non-union surgery [33], emphasizing its versatile utility across diverse orthopedic applications. A single construct delivers the essential elements required for bone healing, addressing both the mechanical and biological aspects of the healing process [1]. Positioned within the host’s vital bone, it connects to the vascular supply and facilitates colonization by host cells that enter the screw’s Haversian system [37]. Incorporation with the host bone begins immediately upon contact, without eliciting inflammatory reactions. The allogeneic bone undergoes physiological remodeling, which prevents destabilization [38].

In addition to achieving bone healing during non-union surgery, the novel treatment has other advantages. First, it reduces the need for additional incisions for autograft harvesting. Second, in successful unions, there is no need for additional operations to remove metal, as metal usage is ideally minimized or eliminated. Third, in the rare cases where non-union persists after revision surgery, the absence of metal is beneficial as the metal itself could potentially cause or exacerbate pain and swelling [39].

Follow-up surgeries, whether for persistent non-union or hardware removal, place pressure on operating room capacity and incur costs for healthcare systems or insurance providers [40,41]. The economic impact extends further, with complex treatment strategies resulting in substantial socioeconomic costs, including lost wages, productivity loss, and compensation for sick leave [40,42,43].

In this study, our aim is to investigate the impact of different surgical treatment methods for non-union of the foot, ankle, and lower leg based on three primary outcomes: (I) bone healing rate (union rate), (II) time to bone healing (time to union)/time to return to work, and (III) clinical complications. Additionally, we will provide a descriptive analysis of radiographic findings of interest. Conventional surgical methods, including those involving metal screws, plates, nails, and staples, both with and without a bone graft, will be assessed alongside the innovative approach to non-union treatment using the Shark Screw^®^. This study also seeks to explore potential benefits associated with the use of the human allogeneic cortical bone screw (Shark Screw^®^) and aims to show the many possibilities where the human allogeneic cortical bone screw can be used.

## 2. Materials and Methods

Approval from the local Institutional Review Boards (IRBs) was received before the study’s commencement, and the reference numbers are as follows: 35-306 ex 22/23 (for Graz), 1146/2023 (for Linz), 1017/2023 (for Vienna), and M2023-25 (for Friesach).

### 2.1. Study Design and Patients

This retrospective multicenter study collected data from four medical facilities in Austria—Graz, Linz, Wien, and Friesach. Clinical and radiographic data were collected from the medical charts of patients who underwent non-union surgery on the foot, ankle, and lower leg between 2010 and 2022. The specific treatment method for non-union was not a selection criterion. Our study included a total of 62 patients, with 34 patients (55%) in the conventional treatment group (treated with metal hardware ± auto/allograft) and 28 patients (45%) in the Shark Screw^®^ treatment group (treated with the Shark Screw^®^ ± metal plate ± auto-/allograft). Patient data are presented in Table 1. The comorbidities included chronic polyarthritis, chronic obstructive pulmonary disease (COPD), diabetes, epilepsy, hereditary motor and sensory neuropathy (HMSN), myelomeningocele, obesity, osteoporosis, previous cancer treatment, psoriasis arthritis, stroke, and vasculitis. Obesity was overrepresented in the conventional group with 10 cases, as reflected by a significant body mass index (BMI), compared to the Shark Screw^®^ group (Table 1).

### 2.2. Inclusion and Exclusion Criteria

To be eligible for inclusion in the study, patients were required to have at least one follow-up visit at least 6 months post-surgery or until bone healing was documented. The assessment of ‘uneventful bone healing’ was assigned when the patient achieved pain-free full weight bearing, and the corresponding radiographs appeared consistent, indicating no implant breakage or signs of loosening. We examined cases that necessitated surgery or revision surgery due to non-union. These cases encompassed non-union after elective procedures (arthrodesis and osteotomy), after surgical fracture treatment, and after conservative fracture treatment (Table 1). We specifically included cases of tibia non-union to provide a comprehensive view of non-union treatment with both methods, offering potential insights into performance under different scenarios (Table 2). We excluded non-unions related to septic conditions (e.g., osteomyelitis), tumor-related cases, and severe post-traumatic cases (e.g., pilon fracture, talus fracture) owing to their complex and often devastating nature and cases that underwent advanced bone reconstruction methods such as vascularized bone grafts, segment transport, or defect bridging.

### 2.3. Surgical Procedures

Surgical procedures vary with the location of the non-union and the performing surgeon. In the conventional treatment of a non-union, the surgical approach aimed to attain stable fixation and compression through the use of metallic screws, plates, nails, or staples, with the use of biological augmentation (auto-/allograft) when deemed necessary [4,13]. After surgery, patients were provided with a cast or splint and instructed to adhere to a non-weight-bearing protocol until consolidation. Intramedullary nailing (for tibia and hindfoot cases) involved two crucial steps: reaming to promote biology and nail insertion for enhanced stability, followed by a postoperative protocol of early weight [44,45].

The Shark Screw^®^ is suitable for stand-alone osteosynthesis or in combination with other implants and auto-/allografts. Successful placement requires sufficient bone for a stable screw–bone interface. The surgical steps include K-wire guided drilling, thread cutting, and, finally, screw insertion (or transplantation). Several surgical procedures were described beforehand in detail for osteotomy fixation [31] and arthrodesis procedures around the forefoot [32,35], TMT2/3 arthrodesis [34], and ankle arthrodesis with defect bridging [36]. Stiff non-unions were treated differently from mobile non-unions. In stiff non-unions, the Shark Screw^®^ was used as a ‘lead structure’ or scaffold, similar to principles applied by other authors; however, they used autologous material [27,28]. The stiff non-union was gently scraped and drilled without destabilizing the rigid situation. Thereafter, Shark Screw(s)^®^ were inserted to bridge the non-union and to reduce the strain [26]. These patients started protected full weight bearing as early as 4 weeks after surgery. In mobile non-unions, the surgical procedure involved the debridement of fibrotic/sclerotic tissue, the preparation of the fusion site, bone end reduction, compression with bone-holding forceps or clamps, temporary stabilization using K-wires, and the placement of Shark Screw(s)^®^. Patients were then immobilized in a cast or splint for 4 to 10 weeks, with the duration tailored to individual patient conditions and the underlying problem.

### 2.4. Statistics

Data are presented as mean ± SD. Due to the non-Gaussian distribution of the data, a non-parametric Kruskal–Wallis ANOVA was used to calculate significant differences for quantitative values. Ordinal values were evaluated with contingency tables using the Chi-square test for significance. A *p*-value < 0.05 was significant with a power > 0.8. Subgroups were smokers. All statistical analyses were performed using Origin Pro statistical software (OriginPro, version 2023; OriginLab Corporation, Northampton, MA, USA).

## 3. Results

In our study, we explored two treatment groups: the conventional treatment, which involved the use of metal hardware (screws/plates/staples/nails) with or without bone graft, and the osteosynthesis treatment with the Shark Screw(s)^®^, optionally combined with a metal plate in some cases.

### 3.1. Clinical Data

In the conventional treatment group, patients underwent diverse implant procedures: 19 with a plate, 8 with a plate and lag screws, 3 with screws, 3 with nails, and 1 with staples. Autografts, utilized in 15 cases, involved local spongiosa (2 cases) and harvested grafts (13 cases from the iliac crest or proximal tibia), resulting in extra skin incisions in 38.2% (13/34). Additionally, allografts were employed in 4 cases (Table 3).

Within the Shark Screw^®^ treatment group, 20 patients exclusively underwent osteosynthesis with Shark Screw(s)^®^. In four cases, existing metal hardware was retained, and in another four cases, an additional metal plate accompanied the Shark Screw^®^ as part of the learning curve. Autografts, sourced from the iliac crest (one patient) and calcaneus (two patients), necessitated an extra skin incision in 10.7% (3/28). Allografts (excluding the Shark Screw^®^) comprised one bone block and seven instances of demineralized bone material (DBM putty, Table 3).

The rate of extra skin incisions for autograft harvest differed significantly between groups, with 38.2% in the conventional treatment group compared to 10.7% in the Shark Screw^®^ group (*p* = 0.02451, Table 3).

Mean follow-up was 23 months for the conventional treatment group and 11 months for the Shark Screw^®^ group, which was statistically significant (*p* = 0.04187, Table 3).

### 3.2. Bone Healing Rate (Union Rate)

In the conventional treatment group, 28 out of 34 cases (82.3%) achieved uneventful bone healing following non-union surgery. An additional two cases (5.9%) showed delayed union and required further interventions (extracorporeal shock wave therapy, metal removal), thereafter progressing to bone healing. The Shark Screw^®^ treatment group achieved uneventful bone healing in 27 out of 28 cases (96.4%).

### 3.3. Time to Bone Healing (Time to Union) and Return to Work

Time to union was achieved uneventfully in the conventional treatment group after 12.9 ± 8.5 weeks, whereas the Shark Screw^®^ group showed a trend toward faster healing, with a mean of 9.4 ± 3.2 weeks (*p* = 0.05061, Table 3). Return to work occurred at after 15.6 ± 17.9 weeks for the conventional treatment group and 9.7 ± 4.4 weeks for the Shark Screw^®^ group. While there is a tendency toward a shorter return to work in the latter group, it did not reach statistical significance (*p* = 0.1123, Table 3).

### 3.4. Examples for Bone Healing

**Figure 1:**
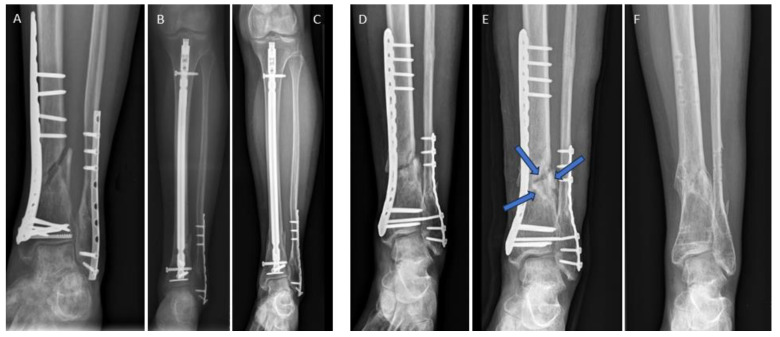
Two comparable cases with distal tibia non-union after fracture surgery. (**A**–**C**) Conventional revision surgery. (**A**) Radiograph showing the non-union of the distal tibia after treatment with metal plates (**B**) Radiograph 4 weeks post-revision with removal of tibial plate, intramedullary reaming, and insertion of a tibia nail. (**C**) Radiograph 24 months after revision. Bone healing was recorded after 12 weeks. (**D**–**F**) Treatment with human allogeneic cortical bone screws. (**D**) Non-union after osteosynthesis with metal plates. (**E**) Revision was performed with three Shark Screws^®^ inserted over the non-union site (blue arrows) acting as strain reducers [26]. (**F**) Bone healing was observed 12 weeks after revision. Plates and metal screws were removed 13 months after successful revision due to hardware-related symptoms.

In Figure 1, we show the revision of a non-union in the distal tibia following failed fracture plating (Figure 1A) with conversion to an intramedullary nail (Figure 1B,C). Successful bone healing was achieved within 12 weeks. Figure 1D–F depicts an alternative treatment employing three human allogeneic cortical bone screws. Introduced via a minimally invasive approach, these screws were inserted after drilling and thread cutting to bridge and stabilize the non-union. This procedure enhanced biological support and reduced strain at the non-union site [26], resulting in uneventful bone healing 12 weeks after intervention. The plate was removed due to persistent irritation thereafter.

**Figure 2:**
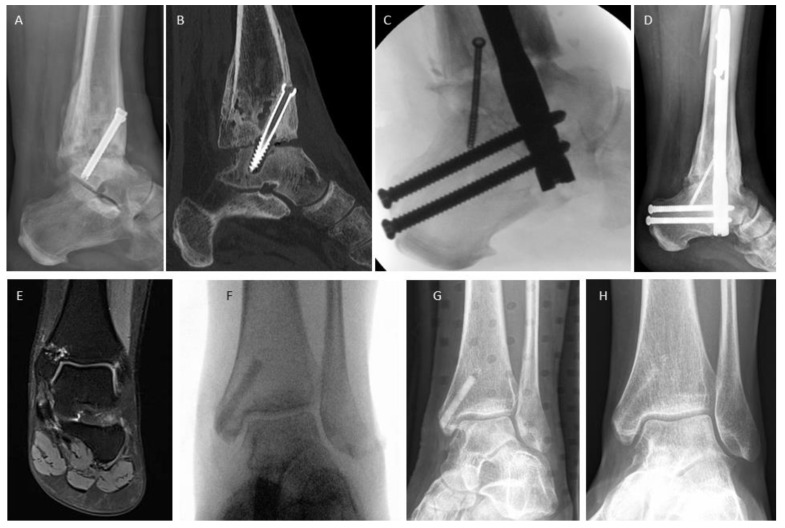
Non-union after ankle surgery. (**A**–**D**) Conventional revision surgery: (**A**) failed screw arthrodesis; (**B**) CT scan depicting the failed arthrodesis; (**C**) intraoperative fluoroscopy following the insertion of a retrograde hindfoot nail; (**D**) radiograph taken 13 months after revision surgery. Bone healing was achieved 10 weeks post-surgery. (**E**–**H**) Treatment with human allogeneic cortical bone screws: (**E**) a case with painful and stiff non-union following medial malleolar osteotomy for the treatment of an osteochondral lesion. The initial osteotomy was secured with metallic screws, later removed due to persistent pain. MRI revealed the underlying issue—the presence of non-union; (**F**) intraoperative fluoroscopy after the insertion of a 4.5 mm Shark Screw^®^ to bridge the stiff non-union; (**G**) 1 month post-revision; (**H**) 11 months post-revision, where the Shark Screw^®^ is barely visible as a result of integration and remodeling. Pain-free full weight bearing with clinical evidence of bone healing was already documented after 6 weeks.

In Figure 2, we demonstrate the treatment of a non-union after the failed screw arthrodesis of an upper ankle joint, which underwent revision with the reaming and insertion of a hindfoot nail (Figure 2A–D). Bone healing was recorded after 10 weeks. Figure 2E–H shows a patient with several years of persistent pain after medial malleolar osteotomy for the treatment of an osteochondral lesion during adolescence. Hardware removal had already been performed, without success. MRI showed signs of non-union that were not detectable on radiographs or CT (Figure 2E), and surgery was performed with one Shark Screw^®^ to bridge the stiff non-union (Figure 2F). Figure 2G reflects the situation 1 month post-surgery. The Shark Screw^®^ is still clearly visible. Bone healing with pain-free full weight bearing was recorded after 6 weeks, and the human allogeneic cortical bone screw was transformed and integrated into host bone after 11 months (Figure 2H).

**Figure 3:**
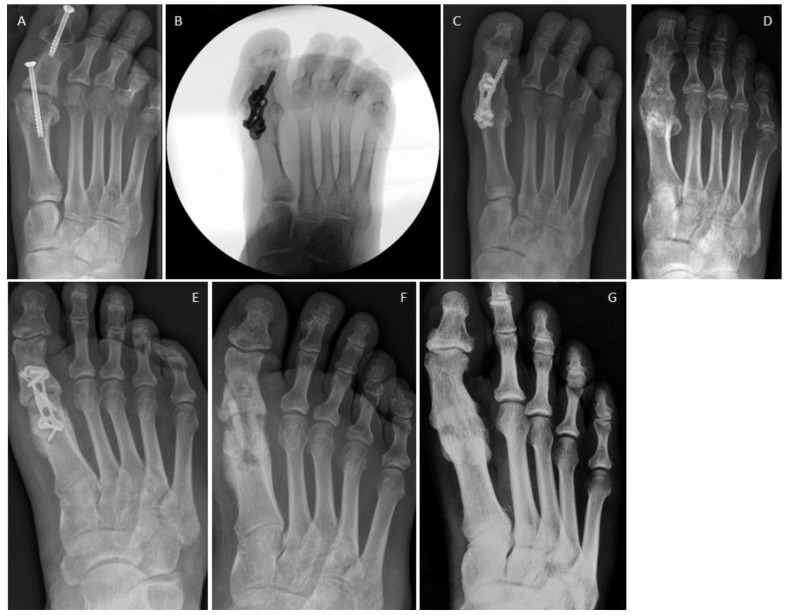
First metatarsophalangeal joints non-unions. (**A**–**D**) Conventional revision surgery: (**A**) Failed arthrodesis at MTP1 (note: the interphalangeal joint was successfully fused); (**B**) intraoperative fluoroscopy after MTP1 revision surgery with a lag screw and a plate; (**C**) radiograph taken 4 months post-revision; (**D**) radiograph captured 12 months after revision and after metal removal, fusion is obvious. Bone healing was recorded after 8 weeks. (**E**–**G**) Revision surgery with human allogeneic cortical bone screws: (**E**) pre-operative radiograph; (**F**) post-operative radiograph, with two Shark Screws^®^ used for revision; (**G**) radiograph taken 12 months post-revision, where the Shark Screws^®^ were hardly visible. Bone healing was recorded after 12 weeks.

In Figure 3, we display an MTP1 joint revision case for both treatment options. Figure 3A–D shows the revision for the conventional (metal) group; Figure 3A illustrates the non-union of the MTP1 joint in a patient treated for symptomatic hallux flexus associated with spastic paresis following a stroke. It is noteworthy that the interphalangeal (IP) screw arthrodesis was successful. The MTP1 joint underwent revision, involving debridement, a lag screw, and a plate. Furthermore, the screw at the fused IP joint was removed (Figure 3B). Bone healing was recorded after 8 weeks, and the screw–plate construct was subsequently removed after 1.5 years due to hardware-related pain (Figure 3D). The bony fusion is well visible. Figure 3E–G presents the non-union of an MTP1 joint following failed plating (Figure 3E). Revision was performed with two human allogeneic cortical bone screws (Figure 3F). Bone healing was recorded after 12 weeks, the Shark Screws^®^ were completely remodeled and transformed to host bone after 12 months (Figure 3G).

**Figure 4:**
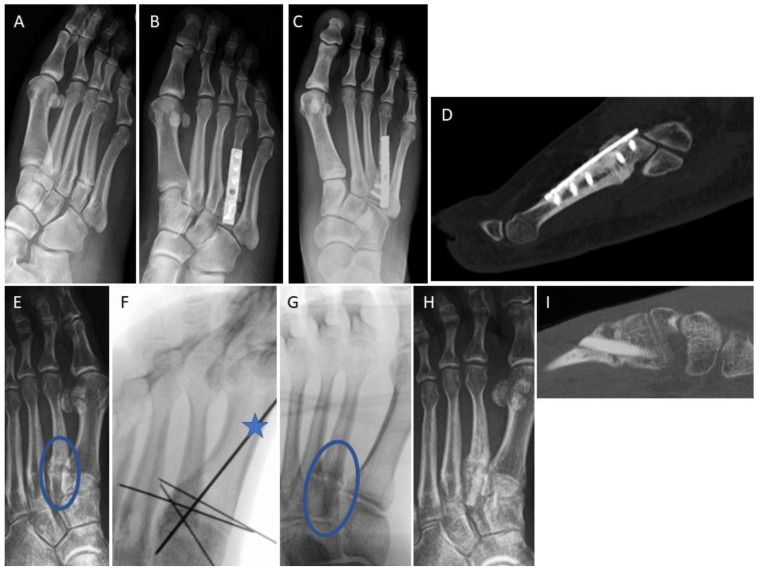
Two comparable cases with stiff non-union at the base of the lesser metatarsals. (**A**–**D**) Conventional non-union treatment. (**A**) A 29-year-old patient with stiff non-union at the base of the 4th metatarsal following a fracture. (**B**) Radiograph two weeks after conventional surgical treatment including the debridement of fibrotic/sclerotic tissue, autologous iliac crest spongiosa transplantation, and plating. (**C**) A weight-bearing radiograph at 3 months confirmed stable conditions. Bone healing was recorded after 8 weeks. (**D**) After 9 years, a CT scan was performed to plan hardware removal surgery due to persistent hardware irritation. (**E**–**I**) Treatment with a minimal invasive dorsal approach using the human allogeneic cortical bone screw. (**E**) A 40-year-old patient with non-union at the base of the second metatarsal (blue ellipse) after a fracture. (**F**) Percutaneous identification of anatomic structures using thin K-wires arranged like goal posts in the intermetatarsal space for orientation. Subsequently, a dorsal 3 cm incision was made, and a 1.6 mm K-wire (blue star) was drilled over the stiff non-union under fluoroscopic control to prepare for K-wire-guided drilling and thread cutting. (**G**) A single 5 mm-diameter Shark Screw^®^ (blue ellipse) was implanted and levelled with a bone saw at the dorsal metatarsal cortex. Fluoroscopy confirmed the accurate transplant position. (**H**) The patient’s recovery progressed well, allowing full weight bearing after 4 weeks, and clinical recovery was achieved. (**I**) A 6 week CT scan showed direct bone contact and the bridging of the former non-union. By this time, the non-union was clinically consolidated, and the patient was pain-free.

Figure 4 depicts a 29-year-old female with the non-union of the base of the 4th metatarsal bone following a fracture (Figure 4A). The patient underwent conventional surgical treatment, which included the debridement of fibrotic/sclerotic tissue, autologous iliac crest spongiosa transplantation, and plating. A follow-up radiograph was taken 2 weeks after revision surgery (Figure 4B). The patient successfully returned to work after 8 weeks. A weight-bearing radiograph at 3 months (Figure 4C) confirmed a stable condition. After 9 years, a CT scan was performed to plan hardware removal surgery due to persistent hardware irritation (Figure 4D). Figure 4E–I represents a 40-year-old female with a non-union at the base of the second metatarsal (blue ellipse) after a fracture (Figure 4E). The non-union was surgically addressed percutaneously using thin K-wires (1.0 mm) placed like goal posts in the intermetatarsal spaces to identify the structure of interest (Figure 4F). We utilized a dorsal minimally invasive approach. Under fluoroscopic control, a 1.6 mm K-wire (blue star) was inserted over the stiff non-union to guide subsequent surgical steps (Figure 4F). The 1.6 mm K-wire was later replaced with a 1.2 mm K-wire to facilitate over-drilling and threading in accordance with recommended Shark Screw^®^ insertion (surgical step not shown). A single 5 mm-diameter Shark Screw^®^ (Figure 4G, blue ellipse) was inserted and levelled with a bone saw at the dorsal metatarsal cortex (Figure 4G). Fluoroscopy confirmed the accurate transplant position. The patient’s recovery progressed well, allowing full weight bearing after 4 weeks, and bone healing was achieved (Figure 4H, 1 month post-surgery). A 6 week CT scan (Figure 4I) showed direct bone contact and the bridging of the former non-union. By this stage, the non-union was clinically consolidated, and the patient was pain-free.

### 3.5. Clinical Complications

Specific complications, involving non-union persistence or the need for further interventions, were noted in 12 patients (35.3%) in the conventional treatment group. In contrast, the Shark Screw^®^ group had significantly fewer complications, with only three patients (10.7%) affected (*p* = 0.02451, Chi-square). Among the three specific complications in the Shark Screw^®^ group, two were linked to precautionary metal implants that were used in combination with the cortical bone screw, necessitating removal—one case due to hardware-related pain and another due to metal plate breakage.

**Figure 5:**
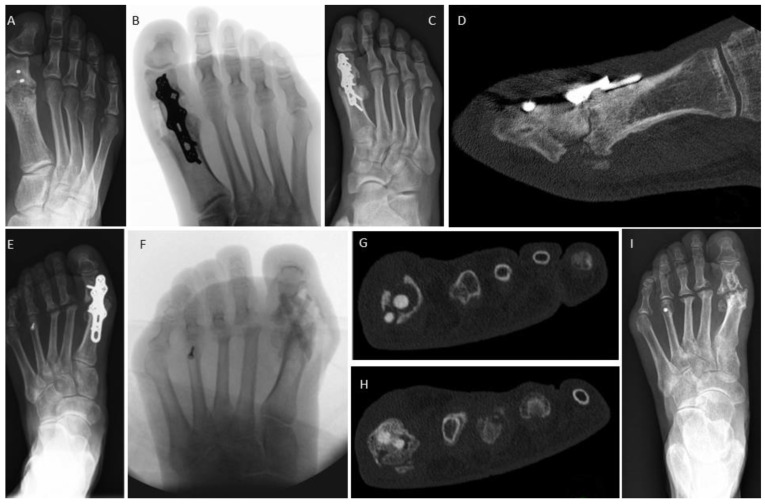
Examples of persistent non-union after first metatarsophalangeal joint revision in both treatment groups. (**A**–**D**) Conventional treatment with structural autograft and metal hardware. (**A**) Pre-operative radiograph after failed MTP1 fusion and partial metal removal. (**B**) Intraoperative fluoroscopy after autologous corticocancellous iliac crest graft and plating. (**C**) Radiograph 5 months post-revision, where union was not obtained. (**D**) CT scan 5 months post-revision, confirming the absence of union. (**E**–**I**) Revision surgery using two allogeneic cortical bone screws. (**E**) A patient with chronic polyarthritis and non-union of the plate arthrodesis at the MTP1 joint, alongside head resection of MT2 and MT3 and Weil osteotomy of MT4. (**F**) Intraoperative fluoroscopy after plate removal and revision with two crossed Shark Screws^®^. (**G**) The CT scan after 4 months reveals poor bone stock and missing interface between the Shark Screws^®^ and the proximal phalanx. Due to the previous surgery and chronic inflammatory process in rheumatic disease, the cancellous bone in the proximal phalanx has completely retreated. It can be seen that the Shark Screws^®^ were in a cavity without contact with the bearing bone (no additional bone graft was inserted). This impeded bone healing at the distal end, resulting in decomposition and the removal of the Shark Screws^®^ in the distal region. (**H**) In the proximal region, the Shark Screws^®^ exhibited a firm connection to the bearing bone of the MT1 head, undergoing successful integration and remodeling. (**I**) Radiograph taken 12 months after revision surgery, where non-union persisted; however, the patient remained pain-free, could stand on tiptoe, and walk without restrictions. The MTP1 joint showed no signs of swelling or hyperemia and exhibited only minimal mobility, with no instability.

The persistence of non-union was seen in four cases (11.8%) in the conventional treatment group, while the Shark Screw^®^ group had one case (3.6%) that was asymptomatic. In contrast, all four non-union cases after failed conventional treatment involving metal hardware had typical symptoms of persistent non-union with pain during weight-bearing and swelling. In Figure 5, examples for persistent non-unions after non-union surgery for both groups are shown. Figure 5A–D shows the revision of an arthrodesis of the MTP1 joint with the removal of formerly used hardware and revision with a cortico-cancellous iliac crest graft and a plate (Figure 5B). Union was not obtained (Figure 5C: Radiograph and Figure 5D: CT-scan) 5 months after revision. The case of persistent MTP1 non-union in the Shark Screw^®^ group is different. Despite the absence of fusion (Figure 5G–I), the patient remained clinically asymptomatic and will be further discussed below.

Delayed union was observed in two cases in the conventional treatment group. A 25-year-old male patient, non-smoker, presented with a non-union following the conservative treatment of an avulsion fracture of the base of the 5th metatarsal. After non-union surgery with two cannulated screws, the clinical symptoms, consistent with delayed union, persisted. However, resolution was achieved following extracorporeal shockwave therapy 36 weeks post-surgery. The second patient was a 74-year-old male patient, a smoker with obesity and COPD, who experienced pain with weight bearing and abnormal swelling, indicative of delayed union. This case ultimately was treated with hardware removal surgery, which led to the resolution of symptoms after 58 weeks.

Metal hardware-related pain (after successful bone healing) was more prevalent in the conventional treatment group with six cases compared to one case in the Shark Screw^®^ group. All these cases were resolved after hardware removal.

Unspecific complications were reported in the conventional treatment group only, with one case involving complex regional pain syndrome (CRPS) and another presenting wound issues.

### 3.6. Radiological Findings of Interest

**Figure 6:**
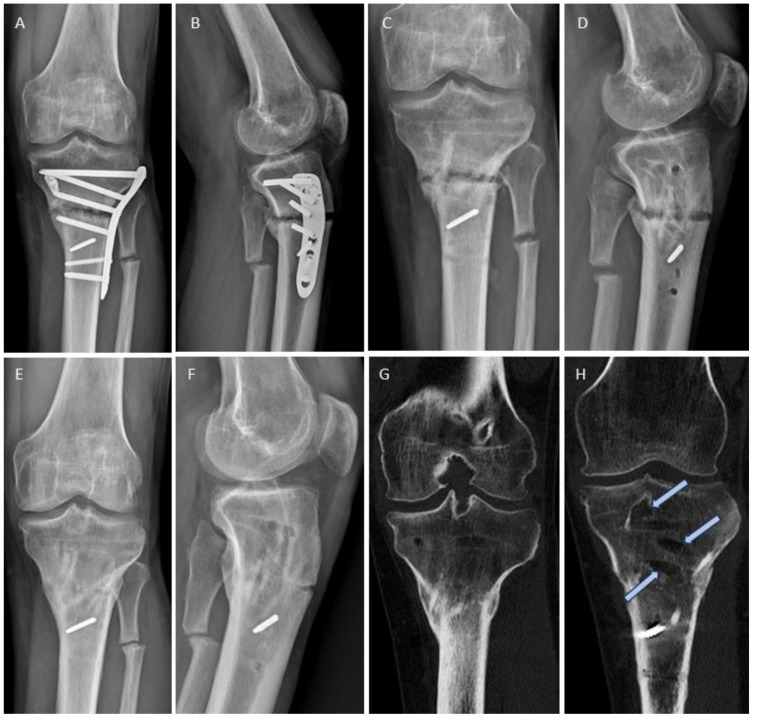
Non-union following a high tibial osteotomy in a 41-year-old smoker in October 2021. (**A**,**B**) Despite extracorporeal shock wave therapy and parathyroid hormone therapy, union was not obtained. The patient experienced severe pain persisting day and night, with limited weight-bearing capacity for short distances. There was a knee extension deficit of 25°. The osteotomy was clinically unstable, opened laterally and medially by approx. 10°, and there was a peroneal lesion with numbness of the lower leg laterally. The patient insisted on metal removal and categorically opposed any future metal treatment. (**C**,**D**) In July 2022, revision surgery with hardware removal, non-union debridement, and axis correction including reclination was performed. The non-union was bridged with an allogeneic bone block and fixed with six Shark Screws^®^. The large bone defects were filled with allogeneic bone paste (DBM putty). (**E**,**F**) Good consolidation of the tibia 14 months after revision. The new bone formation was supported by the bone structure of the allogeneic bone screw, which facilitates cell migration and revascularization. The Shark Screws^®^ have become nearly imperceptible. Bone healing was obtained after 16 weeks. (**G**,**H**) CT scans 15 months after revision. The Shark Screws^®^ have undergone complete remodeling, making them indistinguishable from the host bone (**G**). However, the bone voids resulting from the removal of metal hardware ((**H**), clear blue arrows) have not yet been filled with new bone, even after 15 months.

In Figure 6, we present the case of a 41-year-old smoker who underwent a high tibial osteotomy (HTO) in October 2021. Despite receiving extracorporeal shock wave therapy and parathyroid hormone stimulation, non-union persisted (Figure 6A,B). The patient experienced severe, persistent pain along with limited weight-bearing capacity for short distances. Clinical examination revealed a 25° extension deficit, clinical instability of the osteotomy opening approximately 10° laterally and medially, and a peroneal lesion causing numbness in the lateral lower leg. In July 2022, revision surgery was performed, including hardware removal, non-union debridement, and axis correction, including reclination. The non-union was addressed with an allogeneic bone block and secured/bridged with six Shark Screws^®^. Large bone defects were filled with allogeneic bone paste (DBM putty, Figure 6C,D). Figure 6E,F demonstrates good consolidation of the tibia 14 months after revision. The Shark Screws^®^ are nearly imperceptible due to remodeling, and union was achieved after 16 weeks. Figure 6G,H shows CT scans, taken 15 months after revision surgery. The new bone formation was supported by the bone structure of the human allogeneic bone screw, which facilitated cell migration and revascularization [37]. The Shark Screws^®^ were remodeled and, hence, indistinguishable from host bone (Figure 6G), whereas the bone voids after removing the formerly used metal hardware were still not filled with new bone (clear blue arrows), even after 15 months. The sclerotic wall shielded healing potential and the voids were too large for cell migration, revascularization, and new bone formation, as the potential for trabecular bone formation is spatially limited [46].

**Figure 7:**
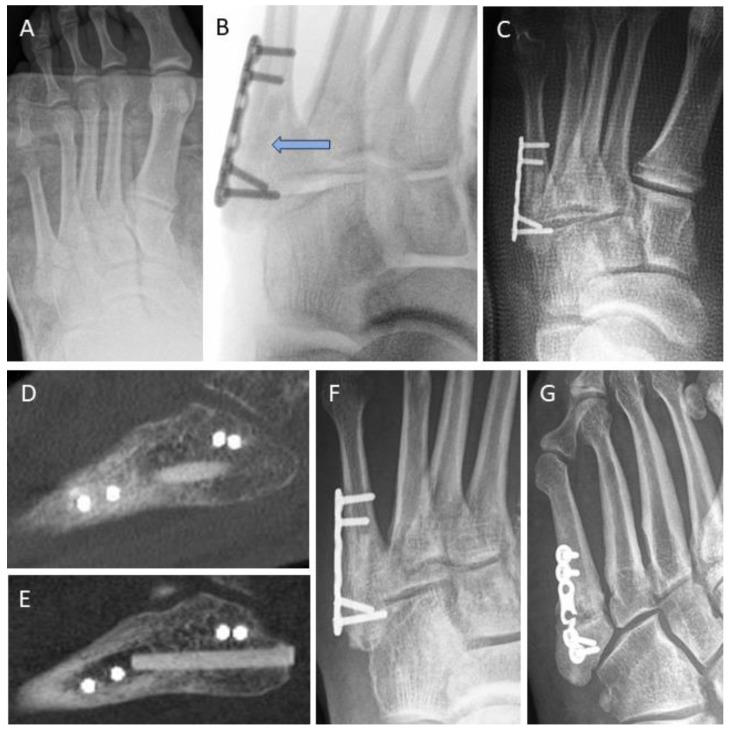
Non-union in a 36-year-old male professional handball player following an avulsion fracture at the base of the 5th metatarsal. (**A**) Conservative therapy that resulted in painful non-union. (**B**) Non-union surgery with a Shark Screw^®^ (blue arrow) and a metallic plate, additionally applied during the experimental phase in the surgeon’s learning curve with the allogeneic bone screw. (**C**) Post-operative radiograph 1 month after surgery. (**D**) CT scan 2 months after surgery. (**E**) CT scan 3 months after surgery showing an intact bone screw and bony union, allowing a return to professional handball. (**F**) Radiograph 4 months after surgery with an intact construct. (**G**) Remarkably, the plate experienced breakage after 5 months, without a refracture of the bone. The patient had only mild symptoms (skin irritation). Hardware removal was deemed necessary.

Metal hardware breakage occurred in two of the four patients from the conventional group with persistent non-union. In contrast, one patient in the Shark Screw^®^ group experienced metal plate breakage despite bony union. Figure 7 depicts the case of a 36-year-old male professional handball player with non-union following the conservative treatment of an avulsion fracture at the base of the 5th metatarsal (Figure 7A). He underwent non-union surgery with a Shark Screw^®^ (blue arrow) and metal plating (as a precaution, Figure 7B). Successful bone healing was observed (Figure 7C–F). CT scans 2 and 3 months after surgery confirmed bone healing and an intact transplant, enabling the patient to return to professional handball (Figure 7D,E). However, the plate experienced breakage after 5 months without a fracture of the Shark Screw^®^ (Figure 7G). The patient had mild symptoms (skin irritation), indicating the need for hardware removal.

Shark Screw^®^ transplant remodeling without radiographic signs of bony union was observed in one case with asymptomatic non-union. A 62-year-old non-smoker with chronic polyarthritis had a prior forefoot surgery involving MTP1 plating, MT2 and MT3 head resection, and Weil osteotomy on MT4. Unfortunately, the MTP1 joint plating resulted in non-union (Figure 5E). The case was revised with two crossed Shark Screws^®^ (Figure 5F) on MTP1. Symptom resolution occurred within two months, but subsequent radiographic evaluations showed gradual transplant resorption and remodeling without radiographic signs of bony union (Figure 5G–I). Intriguingly, at the 1 year follow-up, the patient remained clinically asymptomatic, with a pain-free and mildly mobile (but not unstable) MTP1 joint non-union. The patient can stand on tiptoe and walk without restrictions and was satisfied with the outcome of the revision surgery. The case will be further analyzed in the discussion.

## 4. Discussion

The most important finding of this study is that the surgical treatment of non-union with the human allogeneic cortical bone screw (Shark Screw^®^) resulted in a high rate (96.4%) of bone healing with a shorter time to bone healing (nearly 6 weeks earlier) and a tendency to return to work faster (6 weeks earlier) when compared to the conventional treatment. The Shark Screw^®^ group had fewer complications, with only one case of persistent non-union recorded.

Functioning as both a screw and a bone graft, the Shark Screw^®^ reduces the need for autograft harvest. Our study highlighted a substantial decrease in extra skin incisions for autograft harvest, dropping from 38.2% to 10.7%. This marks a noteworthy absolute risk reduction of 27.5% in experiencing donor site morbidity.

Furthermore, the material’s characteristics help prevent complications associated with metal hardware, eliminating the need for subsequent surgeries for hardware removal [6,47,48].

The conceptual strength of the Shark Screw^®^ lies in its ability to address both stability and biology through a singular transplant without relying on metal hardware or autograft harvest. This dual functionality is unmatched by any other material, particularly one that integrates with the recipient bone (host bone) in harmony with its biology. As Elliott et al. [1] described, the tissue that forms in and around a fracture should be considered a specific functional entity [1]. This ‘bone-healing unit’ produces a physiological response to its biological and mechanical environment, which leads to the normal healing of bone [1], which was confirmed for the human allogeneic cortical bone screw [37]. The Shark Screw^®^ forms a bone-healing unit with the surrounding bone that provides constant exchange [37]. Furthermore, the bone screws bridge the non-union, reducing strain [49]. In Figure 6, the visual difference in new bone formation is evident when employing the human allogeneic cortical bone screw compared to leaving the void empty (after implant removal). The bone allografts enabled bone restitution within a year (Figure 6G,H).

The Shark Screw^®^ group exhibited a 96.4% union rate, surpassing the 82.3% observed in the conventional treatment group. These outcomes align with established literature. Levine et al. [50] reported a 91% union rate (21/23 patients) and an 80% patient satisfaction rate after ankle non-union revision [50]. Easley et al. [51] showed a union rate of 89% (40/45 patients) in ankle revision arthrodesis, with cases of persistent non-union leading to transtibial amputation [51]. Anderson et al. [52] documented an 85% union rate in revision ankle arthrodesis after several procedures in some patients, and the authors reported amputations in cases of infected non-unions, disability, and severe chronic pain [52].

For the midfoot, Hamilton et al. [6] reported an 82% success rate after non-union revision for Lapidus (TMT1) arthrodesis, employing a bone block and autologous bone grafting, along with fixation using screws or a combination of screw and plate fixation [6]. Grambart and Reese [27] illustrated successful examples of a trephine procedure for tarsometatarsal non-union [27]. O’Connor et al. [7] described a 77% success rate after revision arthrodesis for non-union in the foot and ankle, identifying neuropathy and prior revision attempts as the highest risk factors [7]. Dekker et al. [53] achieved union in 83% of patients in revision non-union surgery using cellular bone allograft in foot and ankle procedures in a population with known risk factors for non-union [53]. In our study, non-union/delayed union cases had a high percentage of co-morbidities at the time of surgery (86%, mainly obesity). Kothari’s group [26] recently introduced an intriguing approach to non-union treatment in long bones. They percutaneously inserted a ‘strain reduction screw’ over the hypertrophic non-union, without further addressing biology via bone grafting or changing existing metal implants. Remarkably, this approach achieved a 91% union rate [26,49].

The Shark Screw^®^ group achieved bone healing in a shorter time (9.4 weeks, *p* = 0.05061) compared to the conventional group (12.9 weeks (3 months), excluding delayed unions) and was significant when including delayed unions (15.1 weeks (4 months), *p* = 0.02146). Notably, delayed union was not recorded in the Shark Screw^®^ group. Reported values for time to union vary by location, with isolated fifth metatarsal Jones fractures treated with intramedullary screws showing a reported time of 13.3 weeks [29]. Long bone non-unions entail a longer healing period, averaging around 6 months [54]. In contrast, a distinctive approach to long bone non-union treatment utilizing percutaneous strain reduction screws achieved bone union in around 4 months [26].

The Shark Screw^®^ group demonstrated a notably shorter return to work at 9.7 weeks compared to 15.6 weeks in the conventional treatment group (*p* = 0.1123). Literature reports for return-to-work times/rates after primary procedures varied: 17.9 weeks after ankle arthrodesis [55]; 85% return after Lisfranc injury arthrodesis [56]; 72% within one year after a long bone fracture [57]; and a South African cohort comprising long bone fractures showed a 70% return rate after 1.5 years [58].

In the conventional treatment group, the rate of persistent non-union, or failed fusion, was 11.8% (four patients). Cases included the MTP1 joint (Figure 5A–D), TMT (Lisfranc) joints, talonavicular joint, and calcaneocuboid joint, with all patients experiencing significant pain and functional disability.

In the Shark Screw^®^ group, the rate of persistent non-union, or failed fusion, was 3.6% (one patient). The case concerned revision for MTP1 in a patient with chronic polyarthritis with two crossed Shark Screws^®^, which did not proceed to bony union. (Figure 5E–I). Due to the inflammatory processes in rheumatic diseases and the effects of the previous operation, the cancellous bone in the proximal phalanx had completely vanished. The CT image (Figure 5G) revealed that the Shark Screws^®^ were situated in a cavity without contact with the host bone (the base of the proximal phalanx). This cavity remained unaddressed with additional bone grafting during the revision surgery. Consequently, due to the lack of contact, bone healing could not occur around the base of the proximal phalanx, leading to the removal and decomposition of the Shark Screws^®^. Conversely, at the other end of the fusion site, the Shark Screws^®^ were securely connected to the host bone (the head of MT1) and were, thus, remodeled and integrated in line with their biology as an allo-transplant (Figure 5H). A potential surgical solution might have involved increased allograft usage and enhanced stability in managing the bone defect at the base of the proximal phalanx. Remarkably, the patient remained pain-free with a minimally mobile joint at the 1 year follow-up, highlighting the success of utilizing bioresorbable osteosynthesis material despite clinical and radiographic non-union.

Hope et al.’s proposition [39] regarding hardware removal and debridement as an alternative treatment for non-union finds resonance in our study [39]. A patient in the conventional group achieved recovery after hardware removal (after 58 weeks). Conversely, the Shark Screw^®^ group exhibited a milder clinical scenario for the case with persistent non-union, given the absence of metal involvement (Figure 5E–I).

Overall, complications are high in non-union revision surgery [59], and this fact is mirrored in primary orthopedic procedures. Primary interventions including ankle arthrodesis, tibia fracture osteosynthesis, hallux valgus surgery, and MTP1 arthrodesis consistently demonstrate substantial complication rates. For instance, a systematic review of 1250 primary ankle arthrodesis cases revealed a concerning 31% overall complication rate [60]. Adverse events, including minor and major revisions after primary ankle arthrodesis, were 18% and 22% in high-quality prospective cohorts [61,62], with others large cohorts showing a 7% to 11% major revision rate [63,64]. Tibia fracture treatments show a 30% reoperation rate across all segments of the tibia, according to a cohort study involving 1371 cases from the Swedish fracture registry [65]. Even elective forefoot procedures, such as hallux valgus, exhibit high complication rates, with up to a 30% reoperation rate due to metal removal and revision surgery [48]. In a revision setting, MTP1 joint re-arthrodesis showed a notable 41% overall complication rate [66]. Our study’s conventional treatment group had a specific complication rate of 35.3% (12/34 patients), including bone healing failure and hardware irritation requiring surgical removal. Moreover, unspecific complications occurred, including one case with complex regional pain syndrome and one case with wound issues. The Shark Screw^®^ treatment group showed a significantly lower overall complication rate of 10.7% (3/28 patients) compared to the conventional group (*p* = 0.02451). This comprised one case each of persistent non-union, metal breakage, and hardware irritation, suggesting the potential ability of the Shark Screw^®^ in minimizing complications compared to conventional treatment. Two out of three complications in the Shark Screw^®^ group were linked to metal implants in non-union surgeries on the proximal fifth metatarsal. In these instances, a Shark Screw^®^ was inserted intramedullarly to bridge the stiff non-union, later reinforced with a plate (Figure 7A–G). Plating was deemed necessary during the surgeon’s learning phase with the novel treatment. Nonetheless, when the bone screw was independently used in other cases within the cohort, successful healing without complications occurred (compare Figure 4E–H).

The conventional treatment group comprised seven patients that underwent surgical hardware removal (six due to irritation after successful healing, one due to delayed union). Notably, the count escalates technically when the four persistent non-union cases, each with inlaying metalwork, undergo revision surgeries, including metal removal. This would result in 32% (11/34) of cases requiring further surgical attention due to ‘problematic hardware’. The application of the human allogeneic cortical bone screw offers a solution to avoid this complication.

In our cohort of non-unions, 37% were smokers, surpassing the general smoking prevalence of 18.4% of the EU population that smoke daily [67]. We observed a delayed time to achieve bone healing in smokers, aligning with prior research [5,8,68]. The Shark Screw^®^ may offer advantages to smokers, potentially reducing time to union compared to conventional treatment (10.4 weeks vs. 18.4 weeks, *p* = 0.0914).

Fracture non-union incidences, drawn from comprehensive epidemiology studies—by Mills et al. [69], Walter et al. [40], and Reeh et al. [25] 2022—reveal non-union rates of 1.88, 2.9, and 2.17 per 100,000 capita for foot injuries and 2.6, 2.8, and 3.33 per 100,000 capita for lower leg injuries [25,40,69]. Overall, the combined non-union incidence for the foot, ankle, and lower leg after fracture stands at 5.23 per 100,000 capita. Foot and ankle fusions were recorded at rates of 20.2 per 100,000 capita in the US in 2006 [70]. In Germany, in 2017, the combined incidence of ankle arthrodesis (isolated and those with adjacent joints) and MTP1 joint arthrodesis was 14.7 per 100,000 capita [71]. Assuming a 20 per 100,000 incidence of foot and ankle fusions with an 8% non-union rate, this extrapolates to an incidence of 1.6 per 100,000 capita of non-union incidences stemming from failed fusions. The calculated incidence of non-unions with 5.23 (post-fracture) + 1.6 (failed fusion) cases sums to a total of 6.83 per 100,000 capita around the foot, ankle, and lower leg. Non-union results in high direct healthcare costs, covering surgery, perioperative care, hospital stay, analgesics, and physiotherapy [41], as well as indirect costs like lost productivity and worker’s compensation [43]. A considerable number of patients do not return to work even after one year [57], adding to the financial impact. Additionally, intangible costs, such as residual disability and pain, are substantial [11]. All strategies that decrease healing time and expedite the return to work contribute to alleviating the financial burden in non-union patients [43]. In particular, indirect costs are the key driver of socioeconomic expenses related to non-unions [43]. The economic impact of our findings is significant. With an average non-union incidence of 6.8 per 100,000 capita in the foot, ankle, and lower leg, our calculation projects 30,464 patients annually in the EU (448 million) and 23,120 in the US (340 million). The use of human allogeneic cortical bone screws indicates a trend toward an earlier return to work. Based on a 50% sick leave compensation of net income, an annual after-tax income of EUR 33,500 in the EU [72], equivalent to an average weekly net income of EUR 644, and considering that non-unions primarily affect patients of working age [9], bringing these patients back to work 6 weeks earlier is forecasted to be cost-effective, potentially saving EUR 58.9 million annually in the EU solely with worker’s compensation. In our study, the bone healing rate with the Shark Screw^®^ treatment for non-union surgery was observed to be 14% higher. While acknowledging a trend (*p* = 0.0814), we conservatively assume and calculate a 10% improvement in the union rate. Applying this to all non-unions requiring surgical treatment, we estimate saving 3046 patients (10%) in the EU and 2312 (10%) in the US from further revision surgery. For foot and ankle non-union surgery in Germany, a mean DRG reimbursement of EUR 4524 was reported, and for the lower leg non-union, a mean DRG reimbursement of EUR 6377 was reported [40]. Let us assume a conservative EUR 5500 scenario for non-union surgery in the EU. Even with this cautious assumption and a 10% reduction in cases (−3046 cases), a projected saving of EUR 16.8 million in the EU is anticipated. In the US, treatment costs for non-union after failed ankle arthrodesis were found to sum up to USD 9683 [73], and for long-bone non-union revision surgery, treatment costs ranged from USD 16,000 to USD 34,000 [42,74]. Assuming a USD 10,000 to USD 25,000 scenario per revision surgery in the US, a 10% reduction (−2312 cases) would result in a saving of USD 23.1 to USD 57.8 million. In our study, in the conventional group, secondary surgery due to hardware problems (excluding persistent non-union) occurred in 7 out of 34 patients (20.6%). Assuming this relatively low rate, we conservatively estimate that 6093 patients in the EU (20%) and 4624 patients in the US (20%) will face problems with hardware that need removal after non-union revision surgery. Metal hardware removal around the ankle has direct costs of EUR 797 to EUR 1113 under EU conditions [75,76], and syndesmotic screw removal has direct costs between USD 939 and USD 3579 in the US [77,78]. Avoiding surgery in these patients, assuming direct costs for removal of EUR 1000 in the EU or USD 2000 in the US, would save an additional EUR 6.1 million in the European Union and USD 9.2 million in the US. Furthermore, several days to weeks of sick leave contribute to indirect costs.

## 5. Conclusions

In conclusion, we present here the personalized treatment of non-union. The Shark Screw^®^ presents a reliable option for treating non-unions in the foot and ankle region, and in selected cases, in the lower leg. The presented data show that the use of the human allogeneic cortical bone screw (Shark Screw^®^) results in a high union rate and a trend toward shorter time to union after non-union surgery compared to the conventional treatment. The return to work tended to be earlier for the Shark Screw^®^ group, while conventional treatment was associated with extra skin incisions for autograft harvest, a higher incidence of metal hardware-related pain, and the need for hardware removal surgeries.

Moreover, the potential for cost savings for both the health system and the patient adds weight to the argument in favor of utilizing the Shark Screw^®^ for non-union surgery. Further studies must be performed to underline these findings.

## 6. Limitations

The retrospective design inherently introduces biases, and the participation of multiple surgeons resulted in variations in surgical protocols, which are not detailed. The study included various causes of non-union, such as mechanical issues (e.g., hardware failure), incorrect prior treatment, and factors like smoking and comorbidities. Additionally, it covered diverse anatomical sites, ranging from the forefoot to the lower leg, each with its spectrum of treatment options and different behaviors for bone healing.

## Figures and Tables

**Table 1:** Patient Data.

	Conventional Treatment (Metal Hardware ± Graft)	Shark Screw^®^ Treatment (Shark Screw^®^ ± Metal Plate)	*p*-Value
Number of patients	34	28	
Age [years]	50.2 ± 15.4	49.8 ± 19.2	0.99993
BMI [kg/m^2^]	29.1 ± 6.2	25.4 ± 4.0	0.01780
Gender [male/female]	18/16	11/17	0.28353
Smoker [yes/no]	12/22	11/17	0.74610
Co-morbidities [yes/no]	19/15	7/21	0.02017
Non-union revision following:			0.26197
Elective surgery (arthrodesis, osteotomy)	17	13	
Surgical fracture treatment	8	3	
Conservative fracture treatment	9	12	

**Table 2:** Anatomic location of non-union.

Anatomic Location of Non-Union			Conventional Treatment (Metal Hardware ± Graft)	Shark Screw^®^ Treatment (Shark Screw^®^ ± Metal Plate)
Number of patients			34	28
Forefoot	1st metatarsophalangeal joint		6	8
	1st metatarsal bone		1	1
	2nd to 5th metatarsal bone	subcapital	3	1
	2nd to 5th metatarsal bone	base	6	8
Midfoot	1st tarsometatarsal joint		2	1
	Tarsometatarsal joints	Lisfranc	3	1
	Navicular-medial cuneiform joint		1	1
	Navicular bone		-	1
	Talonavicular joint		4	-
	Calcaneocuboid joint		1	-
Ankle	Medial malleolus		-	1
	Lateral malleolus		3	-
	Lateral and medial malleolus		1	1
	Talocrural joint		1	-
Lower leg	Tibia	distal	1	-
	Tibia	shaft	1	3
	Tibia	proximal	-	1

**Table 3:** Clinical data and outcome.

	Conventional Treatment (Metal Hardware ± Graft)	Shark Screw^®^ Treatment (Shark Screw^®^ ± Metal Plate)	*p*-Value
Number of patients	34	28	
Metal hardware use [n]	34 (*per definition*)	4 (in combination)	-
Autograft use [n (%)]	15 (44.1%)	3 (10.7%)	0.00746
Harvested from surgical site	2 (5.8%)	0	
(And mixed with bone substitute)			
Harvested elsewhere	13 (38.2%)	3 (10.7%)	0.02451
(Extra skin incision required)			
Iliac crest	11	1	
Proximal tibia	2	0	
Calcaneus	0	2	
Allograft use			
Structural [n]	2	28 ^#^	
Demineralized bone matrix [DBM] [n]	2	7	
Follow-up time [weeks (mean ± SD)]	97.2 ± 118.9	47.4 ± 47.1	0.04187
Bone healing [n(%)]			
Achieved after non-union revision surgery	28 (82.3%)	27 (96.4%)	0.08140
Not achieved (= persistent non-union)	4 ^A^ (11.8%)	1 ^B^ (3.6%)	
Achieved after further intervention (= delayed union)	2 ^C,D^ (5.9%)	0	
Time [weeks (mean ± SD)]			
To bone healing (uneventful)	12.9 ± 8.5 (n = 28)	9.4 ± 3.2 (n = 27)	0.05061
To bone healing (including delayed union)	15.1 ± 12.3 (n = 30)	9.4 ± 3.2 (n = 27)	0.02146
Non-smoker	13.3 ± 10.6 (n = 19)	8.7 ± 3.3 (n= 16)	0.41739
Smoker	18.4 ± 14.7 (n = 11)	10.4 ± 2.8 (n= 11)	0.09140
To return to work	15.6 ± 17.9 (n = 30)	9.7 ± 4.4 (n = 25)	0.11230
Clinical complications			
Specific complications [n/(%)]	12 (35.3%)	3 (10.7%)	0.02451
Persistent non-union	4 ^A^	1 ^B^	
Further interventions (overall)	8	2	
Extracorporeal shockwave therapy [ESWT]			
Due to delayed union	1 ^C^	0	
Metal hardware removal surgery			
Due to delayed union	1 ^D^	0	
Due to hardware-related symptoms	6	1 + 1 ^F^	
Unspecific complications [n]	2	0	
Complex regional pain syndrome [CRPS]	1	0	
Wound issues	1	0	
Radiographic findings of interest [n]			
Shark Screw^®^ remodeling without bone healing	*not applicable*	1 ^B^	
Metal hardware breakage	2 ^E^	1 ^F^	

^#^ All 28 patients received one or more Shark Screw(s)^®^, with one additional bone block administered to one patient. ^A^ Non-union persisted in the MTP1 joint, TMT (Lisfranc) joints, talonavicular joint, and calcaneocuboid joint in the conventional treatment group. ^B^ Non-union persisted in the MTP1 joint in one revision case where two crossed Shark Screws^®^ were used. ^C^ Extracorporeal shockwave therapy led to resolution of symptoms in one patient after 36 weeks. ^D^ Metal hardware removal surgery led to resolution of symptoms (pain with weight-bearing) in one patient after 58 weeks. ^E^ Metal hardware breakage was observed in two of the four persistent non-unions in the conventional treatment group. ^F^ Metal plate breakage occurred in a single case, 5 months after treatment with Shark Screw^®^ and neutralization plating for a 5th metatarsal base non-union. Bone healing (confirmed via CT) preceded the plate breakage. Hardware removal was indicated due to professional sport requirements.

## Data Availability

Data are available as Supplemental Data.
